# Communication in Home Care: The Experiences of Formal Caregivers in Communicating with Persons Living with Dementia

**DOI:** 10.1093/geroni/igaa057.3312

**Published:** 2020-12-16

**Authors:** Pabiththa Kamalraj, Marie Savundranayagam, J B Orange, Marita Kloseck

**Affiliations:** Western University, London, Ontario, Canada

## Abstract

There is limited literature on formal caregivers’ communication with persons living with dementia (PLWD) in home settings. Most research comes from studies of long-term care home settings or informal home care contexts. Yet, there are expected needs and rising demands for formal caregiver support within home care. The aim of this study was to understand better the lived experiences of personal support workers (PSWs) regarding their communication with PLWD in home settings. A hermeneutic phenomenological approach guided this research. Semi-structured interviews were conducted with 15 PSW participants. Three major themes were identified through thematic analysis: (1) challenged by dementia-related impairments; (2) valuing communication in care; and (3) home is a personal space. PSWs experienced difficulties in their communication with PLWD despite recognizing the importance of communication in providing optimal home care. This suggests that while PSWs possess good intentions, they do not possess the skills necessary to ensure effective interactions. Dementia-specific education and training are recommended to improve PSWs’ communication skills and to enhance quality of care. Findings highlight further the uniqueness of the personal home space itself on PSWs experiences with communication. Aspects of the home care environment can enable, but also complicate, successful communication between PSWs and PLWD. Consequently, findings also have implications for family members of PLWD and home care employers regarding optimizing practice and improving care.

